# HIV Risk and Interest in Preexposure Prophylaxis in Justice-Involved Persons

**DOI:** 10.3201/eid3013.230739

**Published:** 2024-04

**Authors:** Ank E. Nijhawan, Zoe Pulitzer, Brynn Torres, Natalie Noreen, Alysse Schultheis, Cynthia Frank, Richard Colon, Ralph Brooks, Randi Proffitt, Jennifer Pankow, Ahrein Bennett, Maverick Salyards, Irene Kuo, Kevin Knight, Sandra A. Springer

**Affiliations:** University of Texas Southwestern Medical Center, Dallas, Texas, USA (A.E. Nijhawan, Z. Pulitzer, B. Torres, N. Noreen);; Yale School of Medicine, New Haven, Connecticut, USA (A. Schultheis, C. Frank, R. Colon, R. Brooks, S.A. Springer);; Texas Christian University, Fort Worth, Texas, USA (R. Proffitt, J. Pankow, A. Bennett, M. Salyards, K. Knight);; George Washington University, Washington, DC, USA (I. Kuo)

**Keywords:** HIV, viruses, HIV risk, hepatitis C, preexposure prophylaxis, PrEP, sexually transmitted infections, STIs, persons who inject drugs, PWID, incarcerated, substance use, justice-involved population, justice system

## Abstract

Preexposure prophylaxis (PrEP) is underused in persons who use drugs and justice-involved persons. In an ongoing randomized controlled trial in 4 US locations comparing patient navigation versus mobile health unit on time to initiation of HIV medication or PrEP for justice-involved persons who use stimulants or opioids and who are at risk for or living with HIV, we assessed HIV risk factors, perceived HIV risk, and interest in PrEP. Participants without HIV (n = 195) were 77% men, 65% White, 23% Black, and 26% Hispanic; 73% reported a recent history of condomless sex, mainly with partners of unknown HIV status. Of 34% (67/195) reporting injection drug use, 43% reported sharing equipment. Despite risk factors, many persons reported their risk for acquiring HIV as low (47%) or no (43%) risk, although 51/93 (55%) with PrEP indications reported interest in PrEP. Justice-involved persons who use drugs underestimated their HIV risk and might benefit from increased PrEP education efforts.

In 2021, 6.7 million persons cycled through United States jails (*1*), 443,700 persons were released from state and federal prisons (*2*), and 3.7 million persons were on probation or parole (*3*). During this transition and while under community supervision, those persons are disproportionately affected by health threats such as drug overdose and increased risk for acquiring HIV, sexually transmitted infections (STIs), and hepatitis C (*4**–**9*). Despite successful interventions focused on medications for opioid use disorder (*10*,*11*), implementation of integrated, evidence-based interventions that include HIV prevention has been limited.

HIV preexposure prophylaxis (PrEP) can reduce HIV acquisition by 99% in persons who have sexual exposures (*12*,*13*) and by 74% in persons who inject drugs (PWID) (*14*). However, a considerable unmet need for PrEP exists in highly affected groups, including PWID and justice-involved persons (*15**–**17*). Justice-involved refers to persons who are currently incarcerated (in jail or prison), have a history of being in jail or prison, or are currently or previously on probation/parole. Indications for PrEP include condomless sex with a partner who has HIV or unknown HIV status, recent bacterial STIs, and sharing injection equipment (*18*), all of which are common among justice-involved persons (*19*,*20*), although studies outside those of persons currently incarcerated are limited. Awareness of PrEP is generally low among currently incarcerated persons, ranging from 4% to 25% (*17*,*21*,*22*). Even among persons who have PrEP indications, HIV risk perception is low (*17**,**21*,*23*).

PrEP is not available in most jails and prisons because sex and drug use are prohibited behind bars and providing PrEP might be viewed as condoning or encouraging those behaviors (*24*,*25*). Despite lack of access to PrEP, data from Arkansas, Connecticut, and Rhode Island identified that many justice-involved persons have indications for and express interest in PrEP; noted barriers include individual costs, access to PrEP care, and concerns about side effects (*21*,*23*,*26*). However, limited data exist about PrEP implementation for justice-involved populations, including those in jails or prisons or under community supervision.

In this study, we measured HIV risk with regard to sexual exposures and substance use and describe HIV prevention needs in a diverse justice-involved population enrolled in an ongoing, multisite, randomized controlled trial. Specifically, we assessed sexual and injection drug use risk for HIV acquisition (and their overlap), current self-reported HIV risk, and PrEP awareness, interest, and preferences.

## Methods

A reliance agreement was executed to enable Texas Christian University (TCU) to be the single Institutional Review Board (IRB) of record for all project sites. All project protocols have been reviewed and approved (IRB# 1920-275). Protocol modifications were communicated to TCU IRB, clinicaltrials.gov (NCT05286879), and participants (when appropriate) by site project coordinators and site principal investigators. Additional protections include obtainment of a Certificate of Confidentiality and review and approval of the study protocol by the Office of Human Research Protections at the Department of Health and Human Services. We obtained written informed consent from all project participants.

This study was a preliminary descriptive analysis of baseline assessments conducted for persons enrolled in a hybrid type 1 effectiveness-implementation randomized controlled trial comparing patient navigation to mobile health unit (MHU) for linking justice-involved persons to community-based HIV and substance use disorder (SUD) prevention and treatment services (*27*). Recruitment across 4 study sites in Texas and Connecticut began March 2022. Potential participants were referred by facility staff in jail, prison, court-mandated drug treatment, parole/probation, and the community on the basis of published processes (*27*). For persons in facilities, staff referred any persons who met eligibility criteria. Eligibility included age >18 years; currently in custody with upcoming release date (30 days), recently (previous 6 months) in custody, or currently under supervision (probation, parole); precustody stimulant or opioid use (previous 12 months); precustody history of condomless sex or injection drug use (previous 6 months); and willingness to learn about PrEP. All participants provided written informed consent and then underwent baseline assessments and randomization.

Baseline assessments were conducted face-to-face by research assistants and included demographics, current custody setting (if applicable), housing status, employment, income, and health insurance (precustody if applicable). Mental health disorders were self-reported, and substance use was assessed by using the TCU Drug Screen 5, including fentanyl (*28*). Risk assessment was conducted by using the HIV Risk Behavior Tool (*29*).

We confirmed HIV status chart review and point-of-care HIV testing (Oraquick Rapid HIV 1/2; Orasure Technologies, https://www.orasure.com) for all persons not known to have HIV. For this analysis, only HIV-negative persons were included. We also assessed history of hepatitis C, hepatitis B, gonorrhea, chlamydia, or syphilis.

We asked participants who tested negative for HIV multiple choice questions about self-reported current risk for HIV (no, low, medium or high risk) (*30*), awareness of and interest in PrEP, and if they had ever been prescribed PrEP. All participants were provided standardized education about PrEP from research assistants. If interested in PrEP, participants were asked about preferences, including oral versus injectable and preferred provider location to receive PrEP. They were also instructed to discuss how to get PrEP with an interventionist (patient navigator/community health worker on MHU). If not interested in PrEP, persons provided reasons they were not interested through short free-form answers for reason not interested and preferred location. PrEP indications included self-reported bacterial STI in the previous 6 months, condomless sex with a partner with unknown HIV status or living with HIV within the previous 6 months, and sharing injection equipment.

We entered data into a centralized REDCap database according to study protocol (*27*). We summarized binary and categorical variables by using frequencies and assessed continuous variables by using means. We conducted data cleaning and analyses by using Microsoft Excel R (https://www.microsoft.com) and SAS Studio (SAS Institute Inc., https://www.sas.com).

## Results

Overall, 195 persons without HIV were included. More than three quarters (77%) identified as cisgender male; mean age was 41.4 years; self-reported race/ethnicity was 65% White, 21% Black, and 26% Hispanic. Most (68%) persons reported unstable or temporary housing; completed high school or less (64%); and were either unemployed or on disability (50%) (Table 1).

**Table 1:** Baseline demographic characteristics of 195 participants without HIV in study of HIV risk and interest in preexposure prophylaxis for HIV-negative justice-involved populations in Texas (Dallas and Fort Worth) and Connecticut (northeast and southeast), USA, March 2022–May 2023*

Characteristic	Value
Sex	
M	150 (77)
F	44 (23)
Sex nonconforming	1 (<1)
Mean age, y (SD)	41 (10.3)
Race	
White	127 (65)
Black	40 (21)
Other/unknown	22 (12)
American Indian	4 (2)
Asian	2 (1)
Hispanic ethnicity	51 (26)
Marital status	
Married	16 (8)
Divorced/separated/widowed	61 (31)
Never married	118 (61)
Men who have sex with men	5 (3)
Injection drug users	67 (34)
Housing	
Homeless/shelter	43 (23)
Single occupancy hotel/residential facility	28 (15)
Staying with family/friends	61 (31)
Rent or own home	55 (28)
Education	
Less than high school	46 (24)
High school/GED	78 (40)
Some college/associates/bachelor/graduate degree	71 (36)
Employment	
Full or part time	90 (46)
Unemployed	87 (45)
Disabled/other	18 (10)
Insurance	
Private	16 (8)
Medicaid only	72 (37)
Medicare with or without Medicaid	6 (4)
Other	21 (11)
None	80 (41)
Annual income, US$	
<2,500	79 (41)
12,500–30,000	48 (24)
30,001–50,000	30 (16)
>50,001–100,000	31 (16)
Receive public assistance	104 (53)
Current controlled setting	99 (51)
Recruitment site	
Connecticut (both sites)	78 (40)
Fort Worth, Texas	42 (22)
Dallas, Texas	75 (38)

*Values are no. (%) except as indicated. GED, general educational development test.

There were 16 cases of self-reported STIs in 14 persons within the previous 12 months; 2 (7%) persons had both gonorrhea and chlamydia. Mental health disorders were common (142/195, 73%), as was SUD; 95/195 (49%) had opioid use disorder and 125/195 (64%) had stimulant use disorder (Table 2).

**Table 2:** Clinical characteristics of 195 participants without HIV in study of HIV risk and interest in preexposure prophylaxis for HIV-negative justice-involved population in Texas (Dallas and Fort Worth) and Connecticut (northeast and southeast), USA, March 2022–May 2023*

Characteristic	No. (%)
Hepatitis C	
>1 y ago	36 (18)
4–12 mo ago	6 (3)
1–3 mo ago	4 (2)
Unknown	2 (1)
Hepatitis B	
>1 y ago	4 (2)
Gonorrhea	
>1 y ago	32 (16)
4–12 mo ago	4 (2)
Unknown	2 (1)
Chlamydia	
>1 y ago	3 (19)
4–12 mo ago	3 (2)
1–3 mo ago	1 (<1)
Unknown	1 (<1)
Syphilis	
>1 y ago	10 (5)
4–12 mo ago	7 (4)
1–3 mo ago	1 (<1)
Unknown	1 (<1)
Mental health	
Any issue	142 (73)
Depression	96 (49)
Anxiety	81 (42)
ADHD	37 (19)
PTSD	63 (32)
Bipolar	46 (24)
Schizophrenia	13 (7)
OUD†	
Mild	4 (2)
Moderate	4 (2)
Severe	87 (45)
Stimulant use disorder (cocaine, methamphetamines)†	
Mild	7 (4)
Moderate	12 (6)
Severe	106 (56)
Polysubstance use	
OUD and stimulant use disorders	55 (28)
OUD and AUD	9 (5)
Stimulant use disorder and AUD	13 (7)
OUD/stimulant use disorder/AUD	10 (5)

*ADHD, attention deficit hyperactivity disorder; AUD, alcohol use disorder; OUD, opioid use disorder; PTSD, posttraumatic stress disorder. †Severity of OUD and stimulant use were based on Texas Christian University drug screen scores: mild, 2–3; moderate, 4–5; severe, >6.

At baseline, the mean number of reported sexual partners in the previous 30 days (before custody if applicable) was 2.9 (SD 14.5). One fifth (20%) reported no sexual partners, 39% reported 1 partner, 20% reported 2 partners, and 21% reported >3 partners. Most reported having sex with someone of the opposite sex, although 5 men reported sex with other men; 2 reported transgender partners. Nearly all (91%) who were recently sexually active reported condomless sex; 111 reported vaginal intercourse and 30 both vaginal and anal sex. Of those reporting vaginal sex, 4/141 (3%) had a sexual partner infected with HIV and 74/141 (52%) had partners with unknown HIV status. Most (120/141, 85%) used drugs or alcohol during vaginal sex. Of those reporting anal sex, 1/30 (3%) reported having a partner infected with HIV and 18/30 (60%) reported partners of unknown HIV status. Most (27/30, 90%) reported drug or alcohol use during sex. In the previous 30 days, 67 (34%) of 195 reported injecting drugs and 29 (43%) of /67 (15% of overall cohort) reported sharing equipment. Overlap in substance use and sexual risk was common; 68 (48%) of 141 reported substance or alcohol use during sex with >1 partner infected with HIV or with unknown HIV status. 

**Figure 1:**
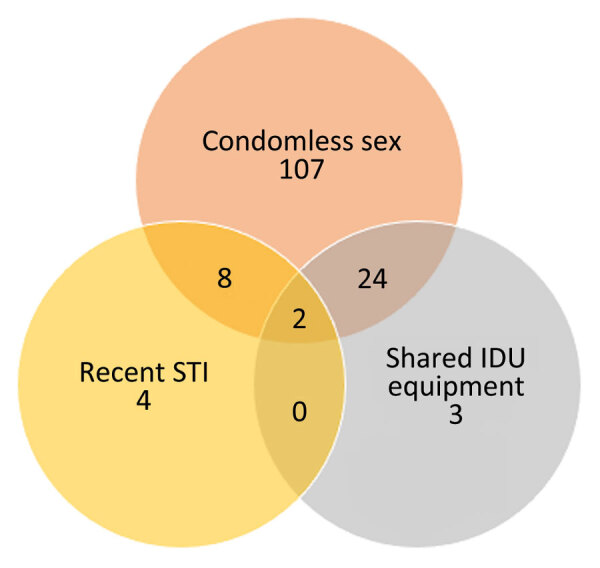
Venn diagram showing indications for preexposure prophylaxis among participants in study of HIV risk and interest in preexposure prophylaxis for HIV-negative justice-involved populations in Texas (Dallas and Fort Worth) and Connecticut (northeast and southeast), USA, March 2022–May 2023. Condomless sex and shared IDU equipment are based on baseline responses with 30-day lookback; recent STI is based on self-report at baseline for STIs diagnosed during the past year. IDU, injection drug use; STI, sexually transmitted infection.

**Figure 2:**
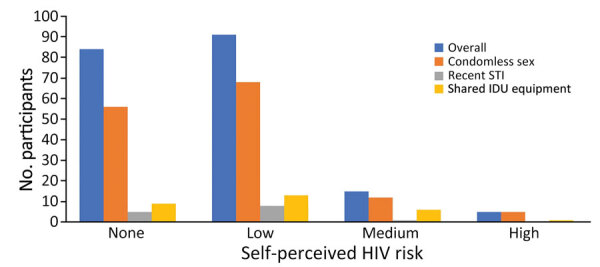
Self-perceived HIV risk overall and by reported risk factors among participants without HIV enrolled in study of HIV risk and interest in preexposure prophylaxis for HIV-negative justice-involved populations in Texas (Dallas and Fort Worth) and Connecticut (northeast and southeast), USA, March 2022–May 2023. Participants answered “what is your current risk for HIV acquisition (no, low, medium or high risk)?” Condomless sex and shared IDU equipment are based on baseline responses with 30-day lookback; recent STI is based on self-report at baseline for STIs diagnosed during the past year. IDU, injection drug use; STI, sexually transmitted infection.

Of 195 participants, 93 (48%) had indications for PrEP (Figure 1), but 90% reported low or no self-perceived risk for HIV, including 13/14 (93%) who had a recent STI and 22/29 (76%) who reported sharing drug equipment (Figure 2). Overall, 113 (58%) of 195 reported being aware of PrEP, 82 (42%) of 195 reported being interested in PrEP, and 1 person had been previously prescribed PrEP. In Texas, 55% were interested versus 23% in Connecticut. Of those recruited while in custody, 53 (53%) of 100 reported interest in PrEP, compared with 29 (31%) of 95 of those recruited from the community. Of those aware of PrEP, 41 (36%) of 113 were interested in taking it, compared with 41 (50%) of 82 who had not heard of PrEP before. Those with PrEP indications were more likely to report interest in PrEP (51/93, 55%) than those without PrEP indications (31/102, 30%; p<0.05).

Of the 82 interested in PrEP, nearly two thirds (62%) preferred injectable PrEP over daily oral PrEP (38%). Preferred locations to receive PrEP were MHU (44%), primary care provider’s office (32%), telemedicine (10%), emergency department (4%), infectious diseases provider (2%), and substance use treatment programs (1%).

Of those not interested in PrEP, 68% believed that they were not at risk for HIV, 11% did not know enough about PrEP, and 9% reported concerns about side effects. Other responses included “do not like taking medicine,” “wanting to focus on primary health needs first,” HIV “was not a death sentence anymore,” and “I’m not gay.”

## Discussion

In a diverse sample of justice-involved persons at risk for HIV who had a history of stimulant or opioid use that were enrolled in an ongoing multicenter randomized controlled trial, participants reported high rates of condomless sex with a partner of unknown HIV status, recent STIs, and sharing injection drug use equipment. Furthermore, approximately half reported overlapping sexual and substance use related risk factors. However, those high rates of HIV risk factors did not correlate with self-perceived risk for HIV; 90% reported low or no risk for HIV, including 93% (13/14) of those who reported recent STIs and 76% (22/29) of those who reported sharing injection drug use equipment. Our findings corroborate others’ findings among persons in jail and prison (*21*,*23*,*26*,*31*), and our study also included community-recruited justice-involved persons.

There are potential reasons for the mismatch between perceived and actual HIV risk in this population. First, when surveyed, persons were often in or recently released from a controlled setting, separated from their sexual and substance use network, and might therefore have assessed their present HIV risk to be lower than their risk when not in custody (*26*,*32*). Second, given the high incidence of HIV among men who have sex with men and messaging from PrEP advertisements and public health campaigns focused on that group, persons in other risk groups (PWID, heterosexual) might believe that they are not at risk for HIV. Third, patients might not be aware of associations between recurrent STIs and HIV (*33*) or the increased HIV prevalence in justice-involved persons and communities disproportionately affected by incarceration (*34*). Our findings reinforce the need for education about HIV risk and PrEP availability in jails, prisons, and community supervision, as well as programs for linkage to PrEP and sexual healthcare.

Only 55% of participants with PrEP indications and 42% overall were interested in PrEP, whereas previous studies reported a range of PrEP interest (23%–90%) (*21*,*23*) among justice-involved groups. PrEP awareness did not correlate with interest, and the main reason for not wanting PrEP was persons believing that they were not at risk, although some also expressed concerns about side effects or not knowing enough about PrEP.

Among those who expressed interest in PrEP, a preference for injectable over oral medications and certain locations for PrEP access (MHU, primary care, or telehealth vs. infectious diseases or substance use treatment clinics) was evident. Some of those preferences (injectable, primary care) might indicate a need for more confidential and less stigmatizing approaches that are also less burdensome to the patient.

Our findings have major implications for HIV prevention initiatives for justice-involved populations, including emphasizing the role substance use might play in sexual risk taking (*35**–**37*), associations between STIs and HIV acquisition, and PrEP indications among PWID. During the time period after custody, recently released persons often have increased substance use (*38*) and increased sexual risk-taking, amplifying the possibility of HIV acquisition (*6**,**7*). Additional multilevel barriers exist to successful PrEP implementation for this group, including competing priorities for meeting basic needs (housing instability, food insecurity), health needs (physical, mental health, SUDs), and other family and legal obligations. Carceral facilities might face competing priorities, limited resources, and lack of experience in implementing PrEP or PrEP education. Furthermore, HIV risk is dynamic in this population (*39*) and requires comprehensive and adaptable healthcare delivery models.

HIV prevention is not limited to PrEP. The role of harm reduction, such as medications for opioid use disorder, syringe exchange, reducing overall substance use, and testing and treatment for STIs, is critical to comprehensive HIV prevention. Although national policies provide a useful framework for reducing HIV incidence (*40*), the omission of SUD screening and treatment as a vital component of HIV prevention will undermine the ability to reduce new HIV infections in the United States, especially for vulnerable populations (*41*,*42*).

Limitations of this analysis include use of cross-sectional baseline data from an ongoing study. Changes over time in HIV risk, attitudes toward PrEP, or PrEP receipt could not be assessed. However, participants will complete follow-up visits at 1, 3, 6, and 12 months, which provides a future opportunity to assess dynamic HIV risk and PrEP uptake. Given the population studied (recent substance use, HIV risk factors, broad criteria for justice-involvement) our findings might not be generalizable to other settings.

In this diverse sample of justice-involved persons who had current or previous substance use, we identified multiple risk factors for HIV acquisition, including sexual and substance use risks. However, participants had low overall self-perceived HIV risk. Less than half were interested in PrEP, and those who were showed preferences for injectable over oral formulations and PrEP delivery preferred through a MHU or primary care, options that might not be widely available. Longitudinal data from this ongoing trial on HIV risk, SUD outcomes, and PrEP interest and initiation in this population will inform future comprehensive HIV prevention approaches.
