# Correction: Vol. 30, Supplement 1

**DOI:** 10.3201/eid3010.C13010

**Published:** 2024-10

**Keywords:** errata, erratum, correction

The vertical axis of Figure 2 was mislabeled in HIV Risk and Interest in Preexposure Prophylaxis in Justice-Involved Persons (A.E. Nijhawan et al.). The article has been corrected online (https://wwwnc.cdc.gov/eid/article/30/13/23-0739_article).

